# The psychological impact of the COVID-19 pandemic on physicians in Puerto Rico: a cross-sectional study after the second wave in 2021

**DOI:** 10.3389/fpsyt.2023.1329427

**Published:** 2024-01-23

**Authors:** Liza C. Sanchez-Plazas, Ricardo García-De Jesus, Karen G. Martinez-Gonzalez, Claudia P. Amaya-Ardila, Israel A. Almodóvar-Rivera

**Affiliations:** ^1^Critical Care Section, Department of Pediatrics, Medical Sciences Campus, University of Puerto Rico, San Juan, Puerto Rico; ^2^Department of Psychiatry, Medical Sciences Campus, University of Puerto Rico, San Juan, Puerto Rico; ^3^Department of Biostatistics and Epidemiology, Medical Science Campus, University of Puerto Rico, San Juan, Puerto Rico; ^4^Department of Mathematical Sciences, Mayagüez Campus, University of Puerto Rico, Mayagüez, Puerto Rico

**Keywords:** anxiety, sleep wake disorders, pandemics, COVID-19, health personnel, organizations

## Abstract

**Introduction:**

Health care providers faced a challenge with the emergence of COVID-19 and its rapid spread. Early studies measuring the psychological impact of COVID-19 on the general population found high levels of anxiety and sleep disorders. The primary goal of this project was to assess the psychological impact of COVID-19 on physicians in Puerto Rico.

**Materials and methods:**

A cross-sectional study of physicians in Puerto Rico was conducted anonymously and electronically from February 2021 through April 2021. The electronic survey included socio-demographic data and 4 self-administered assessment tools (Generalized Anxiety Disorder-7, Perceived Stress Scale-10, Pittsburgh Sleep Quality Index and COVID-19 Organizational Support) for anxiety, perceived stress, sleep disturbances, and organizational support during the COVID-19 pandemic.

**Results:**

A total of 145 physicians completed the survey, with a female predominance of 53.5% and a majority practicing in the San Juan metropolitan area (50.3%). Mild anxiety symptoms were reported in 26.9% of physicians, and 33.8% had moderate to severe anxiety symptoms. Moderate to high perceived stress was found in 69.9% of participants, and women reported statistically significantly higher levels of anxiety symptoms (8.84 ± 5.99; *p* = 0.037) and stress (19.0 ± 6.94, *p* = 0.001). The Pittsburgh Sleep Quality Index reported 67.9% of physicians with global scores associated with poor sleep quality. Assessment of perceived organizational support found a high perception of work support (65.7%) but low perception of personal support (43.4%) and risk support (30.3%). A correlation analysis found a negative correlation for work and personal support, but a positive correlation for risk support, all statistically significant.

**Conclusion:**

COVID-19 had a lasting psychological impact in health care providers in Puerto Rico a year after the beginning of the pandemic. Our data supports the importance of organizational support and its correlation with the development of anxiety. It is thus essential to develop strategies to identify individuals at risk of experiencing psychological disturbances and to provide effective support for medical professionals during medical emergencies for their well-being and optimal delivery of patient care.

## Introduction

Health care providers (HCPs) faced a challenge with the emergence of the novel COVID-19 virus and its rapid spread around the world (1). In late 2019, this highly contagious virus caused the collapse of numerous health care facilities in many countries, with fatal consequences and the uncertainty of a treatment that was merely experimental (2). Physicians are known to work under a lot of pressure and stress based on the nature of their jobs: saving lives. Even without taking into account external conditions, HCPs are at risk of many emotional and psychological consequences due to occupational stress. The unprecedented levels of stress and distress physicians are experiencing are putting them at risk of dissatisfaction with their career and professional burnout, depression, substance use and misuse, and even suicide (3). An Italian study by Epifanio et al. assessed the relationship between burnout and hopelessness in healthcare workers impacted by work related stress during the COVID 19 pandemic. They hypothesized burnout was an important risk factor for the development of hopelessness which has been associated with other psychological conditions such as depression and suicide. The study reported a significant positive correlation between hopelessness in each burnout dimension (4). During the pandemic, high stress levels in physicians (30%) were reported by Linzer et al. in the USA (5). During the COVID-19 pandemic, 15.6% of physicians in Turkey reported moderate stress levels, 10.4%, severe stress levels, and 5.0%, extremely severe stress levels (6).

Studies from China measuring the psychological impact of COVID-19 on the general population conveyed high levels of anxiety (35%) and sleep disorders (18%) (7). In medical staff working in a tense setting and at high risk of infection due to constant exposure, the psychological impact of a pandemic will likely be magnified. A meta-analysis including 13 studies published in 2020 by Pappa et al. presented a pool prevalence of anxiety of 23.2% and depression rate of 22.8% among health care workers during the COVID-19 pandemic (8). Another study in Iran reported high levels of anxiety (28%), depression (30%), and distress (20%) among HCPs (9). A national survey in the USA revealed that 31% of HCPs were experiencing mild symptoms of anxiety but that almost 33% presented with clinically significant anxiety during the COVID-19 pandemic (10). Ara et al. reported in a cross-sectional study in Bangladesh a 60.3% of anxiety symptoms among healthcare professionals during the beginning of the COVID-19 pandemic (11). Other causes of anxiety among HCPs included concerns about the availability of resources and personal protection equipment (PPE), increased workload, fear of infection, fear of infecting family members, poor access to rapid testing, ethical dilemmas, and (lack of) organizational support, to mention several (12).

In addition to the psychological impact of the pandemic, the development of sleep disorders can aggravate the situation. Insomnia is the most frequent sleep disorder, and as defined by the DSM-5, individuals can experience recurrent poor sleep quality, causing distress or impairment in functioning. Insomnia and anxiety disorders can concomitantly affect individuals, triggering significant impairment and disability (13). Individuals with insomnia are 9.8 times more likely to have depression and 17.35 times more likely to have clinically significant anxiety (14). These findings highlight the health and psychological consequences insomnia can have on HCPs exposed to traumatic and stressful circumstances.

Some countries have also endured other stressful circumstances, concomitantly. In Puerto Rico, the first cases of COVID-19 were reported in March 2020. Prior to the pandemic, Puerto Rico had already been facing economic difficulties and a lack of resources. This situation was further aggravated by the catastrophic hurricanes Maria and Irma in September 2017, and subsequently an earthquake swarm that plagued the island from December 2019 well into 2020. A study published in 2019 revealed that around 27% of Puerto Ricans presented anxiety symptoms after hurricane Maria (15). Moreover, a 50% increase in the number of calls received by mental health emergency lines in Puerto Rico was seen during the pandemic. Of those calls, 40% concerned issues related to the pandemic (16). Together, these factors contributed to limiting the infrastructure and the availability of resources to manage crises, leading to higher risks of psychological consequences over time.

Furthermore, organizational support plays an important role in addressing the concerns and fears of HCPs during difficult times, such as a pandemic. Adequate organizational support is linked to lower levels of anxiety and higher levels of life satisfaction (17). Lecca et al. concluded in their literature review the role of having a poor supportive work climate in the development of cardiovascular diseases, depression, suicidal thoughts and psychological wellbeing (18). It is critical for government and health care agencies to implement policies and develop research to protect the psychological well-being of health care workers in every country.

The primary goal of this project was to assess the psychological impact of the COVID-19 pandemic, specifically in terms of anxiety, stress, and sleep disorders, on Puerto Rican physicians after already having endured repeated crises. Also, we aimed to evaluate physicians’ perceptions of organizational support (or the lack thereof) received during the pandemic and its association with anxiety, stress, and sleep disorders. This approach will allow us to develop support strategies based on the specific needs of physicians to prevent the development of serious psychological disorders that could affect their well-being, and, as a result, the care that they deliver to their patients.

## Materials and methods

### Study design

The study had a cross-sectional survey design and was conducted (February through April of 2021) anonymously and electronically including physicians from Puerto Rico. Physicians that were actively working during the pandemic were the population of interest. To accomplish the recruitment, the Puerto Rico College of Physicians and Surgeons (PRCPS) server was used to send physicians invitations to participate in the study. In Puerto Rico, by law, every physician has to be a member of the PRCPS. Using their server every physician registered in the above-named organization was invited to participate in the study. After confirming consent, physicians were asked if they were actively practicing medicine during the COVID-19 pandemic. If the answer was No, the survey ended and automatically excluded the participant from the study. The study was reviewed and approved by the Institutional Review Board of the University of Puerto Rico, Medical Science Campus (protocol B2190620).

### Assessment tools

The email sent to the participants included a sociodemographic survey and 4 self-administered assessment tools. The sociodemographic survey and the assessment tools were administered in English. The sociodemographic survey included such variables as age, sex, marital status, medical specialty and subspecialty, years practicing, and type of workplace (hospital, outpatient). Also, questions related to COVID-19 and recent natural disasters were included, probing such topics as providing direct care to COVID-19 patients, having access to PPE, receiving training for COVID-19 management and protection, and whether a hurricane or hurricanes and/or an earthquake or earthquakes had affected the participant’s practice and/or private property, among others. Four assessment tools that had been validated in the general population, including physicians, were administered to evaluate generalized anxiety disorders, perceived stress, sleep disturbances, and organizational support in the participants.

The Generalized Anxiety Disorder 7 (GAD-7) is a 7-item instrument intended to assess the presence of the symptoms of generalized anxiety disorder, as characterized by the DSM-5 (19). Each item is rated in a 4-point Likert-type scale of frequency, ranging from a minimum of 0 (not at all) to a maximum of 3 (nearly every day). Total scores may range from 0 to 21. The total scores are categorized into 4 severity groups, ranging from minimal (0–4), to mild (5–9), to moderate (10–14), to severe (15–21). The internal consistency of the GAD-7 was reported by Spitzer et al. as excellent with a Cronbach’s alpha =0.92. Test–retest reliability was also described as good with an intraclass correlation = 0.83. Comparison of scores derived from the self-report scales with those derived from the mental health professionals administered versions of the same scales yielded similar results with an intraclass correlation = 0.83, indicating a good procedural validity. At a cut point of 10 or greater sensitivity and specificity exceed 0.80, with an optimized sensitivity (89%) and specificity (82%).

The Perceived Stress Scale (PSS-10) is the most widely used psychological instrument for measuring the perception of stress (20, 21). It is a 10-item instrument; each item is rated using a 5-point Likert-type scale of frequency with 5 response possibilities: never, almost never, sometimes, fairly often, and very often. The scores can range from 0 to 40. The total scores may be categorized in 3 groups, ranging from low perceived stress (0–13) to moderate perceived stress (14–26), to high perceived stress (27–40). Following Campo-Arias et al.’s study, scores equal to or higher than 25 were considered to indicate high perceived stress (in this case, associated with COVID-19) (22). The PSS-10 was derived by eliminating the four items with the lower factor loadings. The 10 remaining items submitted for factor analysis procedures, all loaded positively on the first factor at 0.42 or above. Two factors emerged with values above 1. Deletion of the four items resulted in an improvement in the total explained variance with 48% for both factors combined and internal reliability with a Cronbach’s alpha = 0.78 (23). On a review of the psychometric properties of the three versions of the PSS in 2012, Lee et al. found that the psychometric properties of the PSS-10 were superior to those of the PSS-14 and PSS-4. The Cronbach’s alpha of the PSS-10 was evaluated at >0.70 in all 12 studies in which it was used. The test–retest reliability of the PSS-10 was assessed in four studies and met the criterion of >0.70 in all cases (24).

The Pittsburgh Sleep Quality Index (PSQI) is used to obtain a summary of the sleep experiences and quality of sleep during the previous month (relative to the time that the survey is taken) (25). The instrument contains a total of 24 items: 19 self-rated questions and 5 questions rated by the bed partner or roommate (if either one is available). Only the self-rated questions are used to obtain the global score as per the instrument administration instructions. The 19 self-rated items are combined in such a way as to form 7 components, each of which is scored from 0 to 3 points. Each individual component assesses a specific feature of sleep. The 7 components are overall sleep quality, sleep latency, duration of sleep, sleep efficiency, sleep disturbances, use of sleeping medication, and daytime dysfunction. The scores for each component are added together to obtain a total score, also termed the global score. A global score greater than 5 is associated with poor sleep quality. Only the global scores were used for our analysis. When describing the psychometric properties of the PSQI, as described by Buysse et al., the seven component scores had an overall reliability coefficient or Cronbach’s alpha = 0.83, indicating a high degree of internal consistency and homogeneity. The mean component-total correlation coefficient was 0.58. Individual items were also strongly correlated with each other, indicated by a reliability coefficient of 0.83. When examined the test–retest reliability, paired t tests for the global PSQI score, as well as the seven individual component scores, showed no significant differences between T1 and T2, with correlation coefficient for global PSQI scores of 0.85 (< 0.001). A global PSQI score > 5 yielded a diagnostic sensitivity of 89.6% and specificity of 86.5% (kappa = 0.75, *p* < 0.001) in distinguishing good and poor sleepers.

Lastly, the COVID-19 organizational support (COVID-OS) instrument was used to measure the amount of organizational support that the participants perceived as having received during the COVID-19 pandemic (17). This instrument was created based on an 8-point framework developed by Shanafelt et al. and explores the sources of anxiety of HCPs during the COVID-19 pandemic (7). Each organizational support item is directly related to a source of anxiety in HCPs based on that 8-point framework. Using a 7-point Likert scale whose replies range from “strongly disagree” (1 point) to “strongly agree” (7 points), the participants are asked to rate the extent to which they agree or disagree with each of 8 statements. The instrument is structured in the form of a 3-factor model based on the item’s contents, with said factors being labeled as work support (items 1, 3, and 7), personal support (items 5 and 6), and risk support (items 2, 4, and 8). The questions in the work-support component are related to whether there were adequate PPE supplies, the availability of COVID testing, and, for workers who were deployed to a high-risk unit, organizational support. The questions in the personal-support component are associated with organizational support in terms of childcare and personal and/or non-childcare-related family needs. The last component, risk support, is focused on the risk of getting infected by COVID and infecting a family member or members, the uncertainty as to whether the organization will take care of the respondent’s needs if that respondent were to get infected, and the lack of access to up-to-date information and communication from the pertinent health care system. High scores in the work and personal risk components are associated with high organizational support. Inversely, high scores on the risk-support component were related to low organizational support; the scores of this component were stratified and then classified as low, moderate, or high organizational support. In the work-support component, scores from 0 to 7 were classified as indicating low, from 8 to 14 as moderate, and from 15 to 21 as high organizational support. For personal support, scores from 0 to 5 were considered low, 6–9, moderate, and 10–14, high. Finally, the scores for risk support were inverted, in that those from 0 to 7 were classified as indicating high organizational support, 8–14, as moderate, and 15–21, as low. The initial study results suggested that a prediction of risk of anxiety and life satisfaction in HCPs during the pandemic could be done based on the results of these three components (17). In Zhang et al.’s study, the personal-support component predicted a lower likelihood of mild anxiety, and the work-support component predicted a lower likelihood of moderate anxiety. This instrument was specifically developed to predict anxiety in HCPs and to assess and monitor the specific support offered to HCPs to mitigate their anxiety and fear while working during the COVID-19 pandemic. During the validation of the COVID-OS by Zhang et al., the analysis of this restructured 3-factor model (work support, personal support, and risk support) showed good confirmatory factor analysis fit indices [χ^2^(17) = 38.22, *p* = 0.002; CFI = 0.95; TLI = 0.91; RMSEA = 0.04]. Ordered logistic regression analyses were performed to examine the predictive validities of COVID-OS on anxiety scores. Results showed that work support (*b* = −0.05; 95% CI = [−0.08 to −0.01]; *p* = 0.012), personal support (*b* = −0.04; 95% CI = [−0.07 to −0.01]; *p* = 0.019) and risk support (*b* = −0.05; 95% CI = [−0.09 to −0.00]; *p* = 0.034) were all negatively associated with anxiety. For the clinical utility of the scale in predicting clinical cases, a ROC (receiver operating characteristic) curve on the predictive ability for anxiety (GAD-7 ≥ 10) was performed resulting in an AUC of 0.61 with a sensitivity of 0.66 and a specificity of 0.56 by the Lin criteria for the three factors.

### Statistical analysis

The Research Electronic Data Capture (REDCap) software was used for the survey and data management. Summaries statistics were calculated to describe the sociodemographic variables of the physicians studied. Frequencies with percentages for categorical variables were performed to assess the prevalences of anxiety and sleep disorders. Univariate analysis and Kendall’s Tau-b correlation analysis were used to estimate relationships within the different variables, including demographic variables, other characteristics, anxiety symptoms, sleep disturbances, stress, and organizational support. All the analyses were evaluated with a significance level of 0.05. Statistical analysis was performed using IBM SPSS Statistics for Windows, Version 28.0 (Armonk, NY: IBM Corp).

## Results

A total of 157 physicians in Puerto Rico agreed to participate in the study (from February through April of 2021). Of those, 6 physicians were automatically excluded from the study because they were not practicing during the pandemic. Another 6 participants were excluded because they did not complete their surveys. Ultimately, 145 physicians completed the electronic survey. The vast majority of the physicians were older than 40 years of age 80%, and there was a noticeable female predominance 53.5%. A total of 77.9% were married or living with a life partner. The majority were practicing in the San Juan metropolitan area with a 50.3%, and 59.3% had more than 15 years of experience practicing medicine (Table 1). Moreover, 71% provided direct care to COVID-19 patients. Only 8.3% had become infected with the virus since the beginning of the pandemic. Of our participants, 85.5% referred to having adequate access to PPE, and 76.6% had received training for COVID-19 management and protection (Table 2). Additionally, the participants also answered questions about the impact of the recent (prior to the pandemic) natural disasters on their medical practices. Around 20.7 and 81.4%, respectively, reported that their practices had been affected by the earthquakes and/or hurricanes (Table 3).

**Table 1:** General description of the sociodemographic characteristics.

		%
Age (years)		
18–40	29	20
>40	116	80
Sex^1^		
Female	77	53.5
Male	67	46.5
Marital status		
Unmarried	21	14.5
Married or living with a partner	113	77.9
Divorced/Widowed	11	7.6
Years of practice		
Fewer than 10 years	35	24.1
11–15 years	24	16.6
More than 15 years	86	59.3
Practice region^2^		
Metropolitan area	73	50.3
North region	25	17.2
South region	12	8.3
East region	18	12.4
West region	21	14.5
Central region	14	9.7
Main practice setting		
Hospital	40	27.6
Outpatient	89	61.4
Both	16	11

N^1^, varies because of missing data. N^2^, varies because some physicians work in more than 1 region.

**Table 2:** COVID-19–related questions (characteristics).

	N	%
Provide direct care to COVID patients		
Yes	103	71
No	42	29
Infected with COVID		
Yes	12	8.3
No	133	91.7
Family member(s) infected with COVID		
Yes	28	19.3
No	117	80.7
Adequate access to PPE		
Yes	124	85.5
No	21	14.5
Training for COVID management and treatment		
Yes	111	76.6
No	34	23.4

PPE, personal protective equipment.

**Table 3:** Natural disaster–related questions (characteristics).

	N	%
Practice affected by earthquake(s)		
Yes	30	20.7
No	115	79.3
Practice affected by hurricane(s)		
Yes	118	81.4
No	27	18.6

### Prevalences of anxiety symptoms, perceived stress, and sleep disturbances of physicians during the COVID pandemic

**Figure 1:**
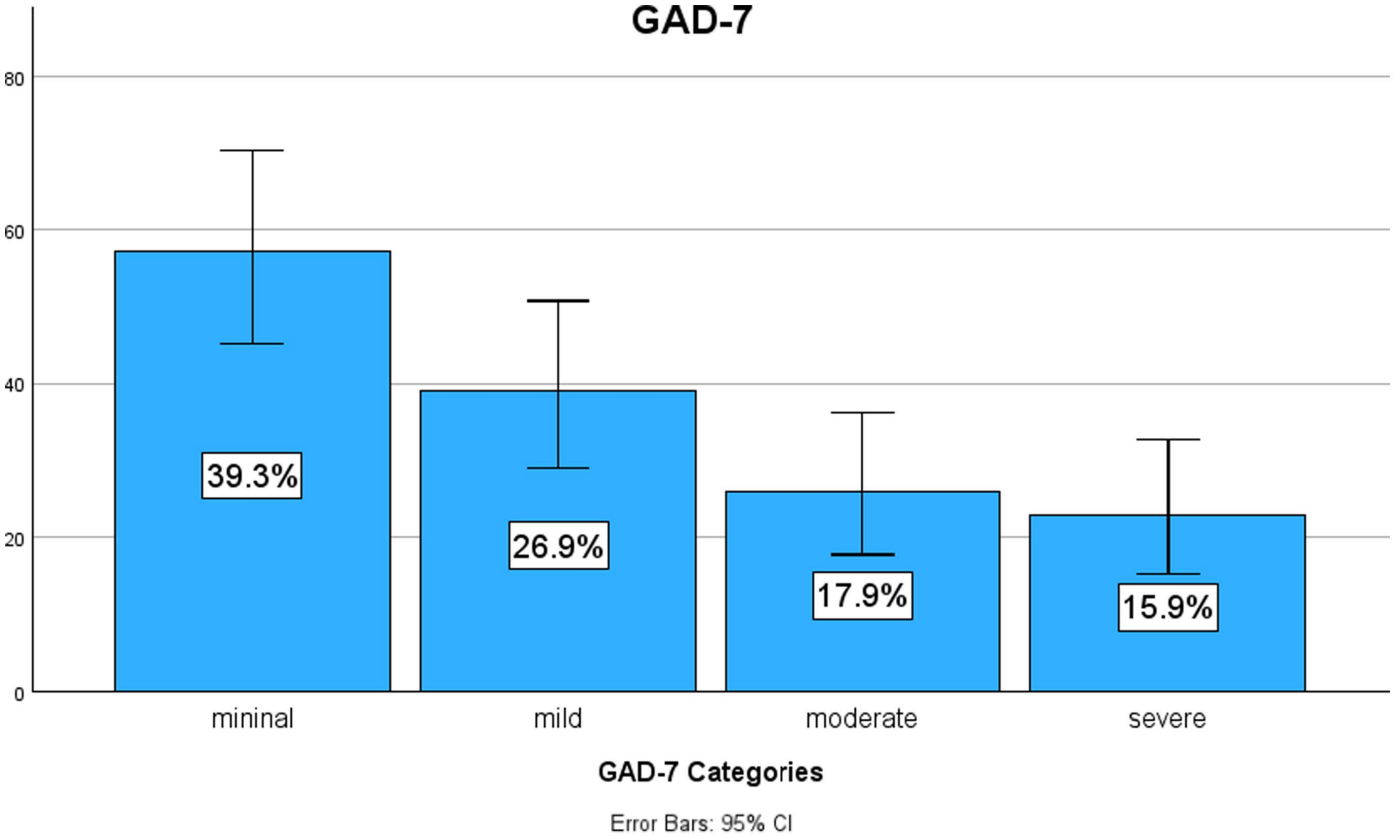
Prevalence of anxiety symptoms. Prevalence of anxiety symptoms in physicians working in Puerto Rico during the pandemic. GAD-7, Generalized Anxiety Disorder 7. Figure by SPSS program.

**Figure 2:**
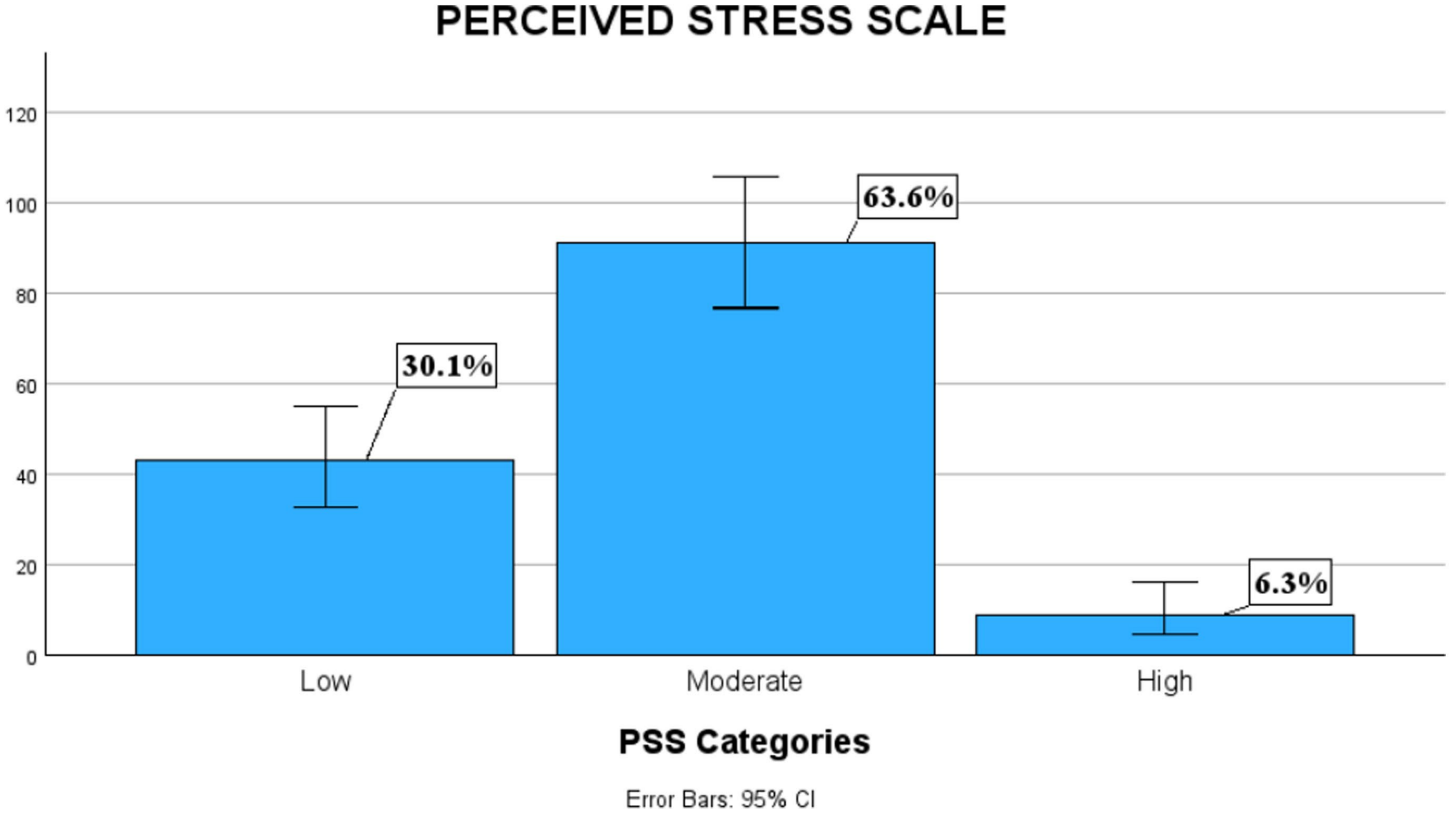
Prevalence of perceived stress. Prevalence of stress in physicians working in Puerto Rico during the pandemic using the Perceived Stress Scale scores. Figure by SPSS program.

Using the GAD-7 tool, we found that 39.3% of the physicians had minimal anxiety symptoms. Nearly 26.9% reported having mild anxiety symptoms, but approximately 33.8% were classified within the moderate or severe anxiety symptom groups (Figure 1). The average anxiety symptom score was 7.82 (SD ± 6.10). However, when assessing perceived stress, approximately 63.6 and 6.3% of the physicians, respectively, reported having moderate or severe stress (Figure 2). The mean PSS score was 17.2 (SD ± 7.08). Higher scores for the GAD-7 and PSS were found in women than in men (Table 3). For these measures, the women physicians reported significantly higher levels of anxiety symptoms with a mean of 8.84 (SD ± 5.99), *p* = 0.037 and stress mean of 19.0 (SD ± 6.94), *p* = 0.001 during the pandemic than did men physicians, in terms of anxiety with a mean of 6.71 (SD ± 6.08) and stress mean of 15.2 (SD ± 6.71) (Table 4). Neither age nor years of experience were found to be statistically significant. Physicians whose practices were affected by earthquakes also had higher scores on the GAD-7, representing statistically significantly higher levels of anxiety symptoms with a mean of 11.1 (SD ± 6.45) compared to those who were not affected by earthquakes with a mean of 6.95 (SD ± 5.71), *p* < 0.001 (Table 5). Also, moderate perceived stress with a mean of 19.5 (SD ± 8.06), *p* = 0.050 was found in physicians affected by earthquakes, close to reaching statistical significance. Moreover, physicians whose practices were affected by hurricanes had mild symptoms of anxiety with a mean of 8.12 (SD ± 6.24), *p* = 0.218 and moderate perceived stress with a mean of 17.21 (SD ± 7.20), *p* = 0.900, but neither issue was statistically significant. High scores for PSS were found in physicians who reported having inadequate PPE with a mean of 20.6 (SD ± 6.71), compared to those who reported having adequate PPE with a mean of 16.6 (SD ± 7.01), *p* = 0.018 (Table 6).

**Table 4:** Univariate analysis for sex and dependent variables.

	Sex						Statistics results			
	Female			Male						
	N^1^	M	SD	N^1^	M	SD	*t*	df^1^	*p-*value	95% CI
Score on GAD-7	77	8.84	5.99	67	6.71	6.08	2.108	142	0.037*	(0.13, 4.12)
Score on PSQI	68	7.42	3.53	54	6.53	3.81	1.333	120	0.185	(−0.43, 2.21)
Score on PSS	76	19.0	6.94	66	15.2	6.71	3.333	140	0.001*	(1.55, 6.10)
Score on COVID-OS/work support	77	14.8	3.87	65	15.3	4.50	−0.750	140	0.454	(−1.91, 0.86)
Score on COVID-OS/personal support	77	6.16	3.37	67	6.88	3.82	−1.222	142	0.238	(−1.89, 0.47)
Score on COVID-OS/risk support	77	13.1	4.03	67	12.7	4.07	0.564	142	0.574	(−0.95, 1.72)

GAD-7, Generalized Anxiety Disorder 7; PSQI, Pittsburgh Sleep Quality Index; PSS, Perceived Stress Scale; COVID-OS, COVID-19 organizational support; M, mean; SD, standard deviation; df, degrees of freedom. *Indicates statistical significance (*p* < 0.05) using independent *t*-test for equality of means. N^1^ and df^1^ vary because of missing data.

**Table 5:** Univariate analysis for practices affected by earthquakes and dependent variables.

	Earthquakes affected practice						Statistics results			
	Yes			No						
	N^1^	M	SD	N^1^	M	SD	*t*	df^1^	*p*-value	95% CI
Score on GAD-7	30	11.11	6.45	115	6.95	5.71	−3.495	143	<0.001*	(−6.59, −1.82)
Score on PSQI	26	7.92	3.62	97	6.79	3.64	−1.410	121	0.163	(−2.72, 0.46)
Score on PSS	30	19.5	8.06	113	16.6	6.71	−1.974	141	0.050	(−5.69, 0.004)
Score on COVID-OS/work support	30	14.2	5.16	113	15.3	3.85	1.076	37.9	0.289	(−0.95, 3.13)
Score on COVID-OS/personal support	30	5.90	3.91	115	6.72	3.56	1.10	143	0.272	(−0.65, 2.29)
Score on COVID-OS/risk support	30	12.5	4.29	115	13.0	3.98	0.563	143	0.574	(−1.17, 2.11)

GAD-7, Generalized Anxiety Disorder 7; PSQI, Pittsburgh Sleep Quality Index; PSS, Perceived Stress Scale; COVID-OS, COVID-19 organizational support; M, mean; SD, standard deviation; df, degrees of freedom. *Indicates statistical significance (*p* < 0.05) using independent *t*-test for equality of means. N^1^ and df^1^ vary because of missing data.

**Table 6:** Univariate analysis for independent and dependent variables.

	COVID patient care						Statistics results			
	Yes			No						
	N^1^	M	SD	N^1^	M	SD	*t*	df^1^	*p*-value	95% CI
Score on GAD-7	103	7.76	6.00	42	7.97	6.41	0.187	143	0.852	(−2.00, 2.42)
Score on PSQI	87	7.36	3.71	36	6.22	3.44	−1.590	121	0.114	(−2.57, 0.28)
Score on PSS	102	17.4	6.96	41	16.7	7.44	−0.529	141	0.598	(−3.29, 1.90)
Score on COVID-OS/work support	103	15.1	4.38	40	15.1	3.60	−0.088	141	0.930	(−1.47, 1.60)
Score on COVID-OS/personal support	103	6.65	3.70	42	6.31	3.49	−0.510	143	0.611	(−1.66, 0.97)
Score on COVID-OS/risk support	103	13.4	4.07	42	11.5	3.65	−2.652	143	0.009*	(−3.35, −0.49)

	Adequate PPE						Statistics results			
	Yes			No						
	N^1^	M	SD	N^1^	M	SD	*t*	df^1^	*p*-value	95% CI
Score on GAD-7	124	7.74	6.18	21	8.33	5.68	0.410	143	0.683	(−2.26, 3.44)
Score on PSQI	106	7.00	3.70	17	7.17	3.41	0.174	121	0.862	(−1.73, 2.06)
Score on PSS	122	16.6	7.01	21	20.6	6.71	2.396	141	0.018*	(0.69, 7.20)
Score on COVID-OS/work support	124	15.4	4.02	19	12.9	4.49	−2.493	141	0.014*	(−4.50, −0.51)
Score on COVID-OS/personal support	124	6.66	3.72	21	6.04	3.13	−0.685	143	0.494	(−2.29, 1.11)
Score on COVID-OS/risk support	124	12.6	4.02	21	14.9	3.67	2.452	143	0.015*	(0.44, 4.15)

	COVID training						Statistics results			
	Yes			No						
	N^1^	M	SD	N^1^	M	SD	*t*	df^1^	*p*-value	95% CI
Score on GAD-7	111	7.78	6.12	34	7.97	6.11	0.156	143	0.876	(−2.18, 2.55)
Score on PSQI	94	6.75	3.65	29	7.93	3.57	1.522	121	0.131	(−0.35, 2.70)
Score on PSS	110	16.6	6.81	33	19.2	7.70	1.826	141	0.070	(−0.21, 5.30)
Score on COVID-OS/work support	111	15.6	4.02	32	13.3	4.21	−2.813	141	0.006*	(−3.90, −0.68)
Score on COVID-OS/personal support	111	6.80	3.60	34	5.73	3.68	−1.501	143	0.135	(−2.47, 0.33)
Score on COVID-OS/risk support	111	12.5	4.16	34	14.0	3.42	1.863	143	0.064	(−0.08, 3.01)

GAD-7, Generalized Anxiety Disorder 7; PSQI, Pittsburgh Sleep Quality Index, PSS, Perceived Stress Scale; PPE, personal protection equipment; COVID-OS, COVID-19 organizational support; M, mean; SD, standard deviation; df, degrees of freedom. *Indicates statistical significance (*p* < 0.05) using independent t-test for equality of means. N^1^ and df^1^ vary because of missing data.

PSQI was used to evaluate seven components of sleep to assess sleep disturbances. Those components consisted of sleep duration, sleep disturbances, sleep latency, day dysfunction due to sleepiness, sleep efficiency, the use of medication to sleep, and overall sleep quality. Over 27% of the physicians had sleep durations of less than 6 h per night, 33.1% had moderate to severe sleep disturbances, 51.1% had poor sleep latency, and 35.2% had high scores for poor overall sleep quality. According to the measure, 67.9% had a global PSQI score higher than 5, which is associated with poor sleep quality. The average PSQI score was 7.03 (SD ± 3.65). On univariate analysis, women scored higher than men in terms of poor sleep quality, with a mean of 7.42 (SD ± 3.53), compared with males with a mean of 6.53 (SD ± 3.81) but this was not found to be statistically significant on independent *t*-test, *p* = 0.185 (Table 4). Physicians whose practice was affected by earthquakes were found to have poor sleep quality with a mean of 7.92 (SD ± 3.62) compared to physicians their practices were not affected with a mean of 6.69 (SD ± 3.64), *p* = 0.163, but this did not reach statistical significance either. None of the other independent variables were found on univariate analysis to be statistically significant.

### Physician-perceived organizational support

**Figure 3:**
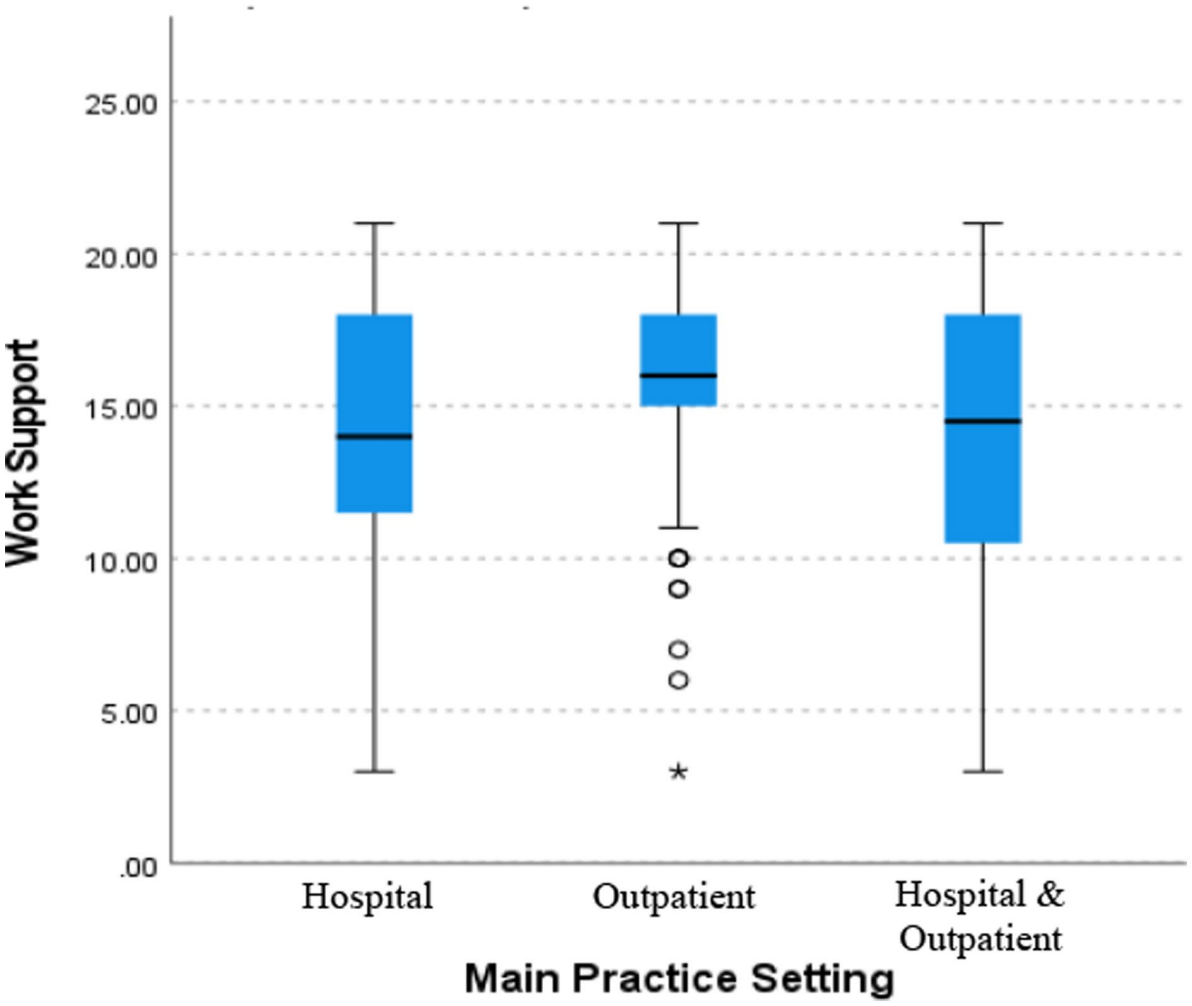
Comparison means for main practice setting and work support. Box plot diagram showing the distribution of the data and sample variability between the main practice setting of the physicians in relation to the dependent variable work support. Figure by SPSS program. ⚬ = outlier; * = far outlier.

The assessment of perceived organizational support during the COVID-19 pandemic was achieved by evaluating the 3 components of the COVID-OS tool, which is based on the 8-point framework developed by Shanafelt and looks at the sources of anxiety in HCPs. Those three components are work support, personal support, and risk support. The scores of the participating physicians were linked to a high perception of work support with a 65.7% but to a low perception of personal support with 43.4% and risk support with 37.2% from their organizations. The average mean scores of the COVID-OS components were 15.1 (SD ± 4.16) for work support, 6.55 (SD ± 3.63) for personal support, and 12.9 (SD ± 4.04) for risk support. When evaluated by univariate analysis and analysis of variance, statistically significant differences in means were found. A One-way analysis of variance was performed to compare the effect of practice setting (hospital, outpatient, or both) on the dependent variables (scores on the GAD-7, PSQI, PSS, and COVID-OS components) (Table 7). When the test of homogeneity of variances was performed, the GAD-7 and work support dependent variables did not meet the homogeneity assumption through Levene test. The Kruskal–Wallis test was performed to determine whether the practice setting affected the GAD-7 and/or work-support scores. The analysis revealed that there was a statistically significant difference in perceived work support [H(2) = 6.918; *p* = 0.03], with higher perceived work support for physicians working in outpatient settings than for those working in a hospital or in both settings. An evaluation of the pairwise comparison of main practice settings revealed that the true differences were between the hospital-based and outpatient-serving groups, with an adjusted significance of *p* equaling 0.054, by Bonferroni correction. Despite a larger range or wider spread of the data in the hospital setting, the outpatient group has a higher median, which equates to a greater perception of work support in that sample (Figure 3). When analyzing the GAD-7 using the Kruskal–Wallis test, no statistical significance was achieved [H(2) = 2.063; *p* = 0.356], with no difference in the medians between practice settings found. Also, using ANOVA test, there was a statistically significant difference in risk-support scores between at least two groups [*F*(2,145) = [3.084]; *p* = 0.025] in physicians working in the hospital, with higher scores in that component (Table 7). In this analysis of Tukey’s honestly significant difference test for multiple comparisons, it was found that the mean values for risk support were significantly different between hospital and outpatient physicians. Higher scores in the risk support component (resulting in a lower perceived risk support) were found in the hospital group when compared with outpatient group (Mean difference: 2.05, *p* = 0.020; 95% CI: 0.26, 3.84). The confidence intervals here, not including zero, also indicate that there was a difference of means between both groups. There was no statistically significant difference between the physicians working in both settings and physicians working in the hospital (*p* = 0.711) or between the physicians working in both settings and physicians working outpatient (*p* = 0.549).

**Table 7:** One-way ANOVA for differences in means between practice settings.

	Practice setting									Statistics results				
	Hospital			Outpatient			Hospital and outpatient							
	N^1^	M	SD	N^1^	M	SD	N^1^	M	SD	F/W	SS	df	MS	*p*-value
Score on GAD-7	40	6.45	4.74	89	8.20	6.42	16	9.18	6.98	2.063		2	0.356 †	
Score on PSQI	33	7.33	3.63	77	7.77	3.79	13	7.76	2.89	0.556	14.9	2	7.49	0.575
Score on PSS	39	15.8	6.18	88	17.4	7.27	16	19.5	7.86	1.561	155.6	2	77.8	0.214
Score on COVID-OS/work support	40	13.9	4.80	87	15.9	3.45	16	13.7	5.11	6.918	20.031*†			
Score on COVID-OS/personal support	40	6.45	4.09	89	6.80	3.47	16	5.37	3.28	1.075	28.4	2	14.2	0.344
Score on COVID-OS/risk support	40	14.3	4.30	89	12.2	3.69	16	13.3	4.55	3.804	119.7	2	59.8	0.025*

ANOVA, analysis of variance; GAD-7, Generalized Anxiety Disorder 7; PSQI, Pittsburgh Sleep Quality Index; PSS, Perceived Stress Scale; COVID-OS, COVID-19 organizational support; M, mean; SD, standard deviation; F, F statistics for ANOVA; W, Kruskal–Wallis statistics; SS, sum of squares; df, degrees of freedom, MS, mean squares. *Indicates statistical significance (*p* < 0.05) using the ANOVA test to compare the means of the comparison groups. N^1^ varies because of missing data. ^†^Indicates Kruskal Wallis test was performed.

Physicians who reported providing care to COVID-19 patients had high scores on the risk-support component of the COVID-OS—which scores were related to low perceived levels of risk support provided by their organizations with a mean of 13.4 (SD ± 4.07)—compared to physicians who did not provide care to COVID-19 patients with a mean 11.5 (SD ± 3.65), *p* = 0.009. Physicians who reported having received training for COVID management and protection had high scores on the work-support component of the COVID-OS—which scores were related to a high perceived levels of work support by their organizations with a mean of 15.6 (SD ± 4.02)—compared to physicians who did not receive training for COVID with a mean of 13.3 (SD ± 4.21), *p* = 0.006. Physicians who referred to having adequate PPE had high scores in the work-support component of the COVID-OS—which scores were related to high levels of work support perceived by their organizations with a mean of 15.4 (SD ± 4.02)—compared to physicians who did not have adequate PPE with a mean of 12.9 (SD ± 4.49), *p* = 0.014 (Table 6). Finally, physicians who referred to having inadequate PPE had higher scores on the risk support component—which scores were related to poor risk support by their organizations with a mean of 14.9 (SD ± 3.67)—when compared to those who had adequate PPE with a mean of 12.6 (SD ± 4.02), *p* = 0.015.

### Associations between organizational support, anxiety, stress, and sleep disorders

Kendall’s Tau-b correlation coefficient was computed to assess the relationship within the variables, considering the COVID-OS components and anxiety, stress, and sleep disorders. There was a negative correlation between work support and stress that was statistically significant (*τ*_b_ = −0.201, *p* ≤ 0.001). Another statistically significant negative correlation was found between work support and sleep disorders (*τ*_b_ = −0.168, *p* = 0.010). Therefore, as work support increased, there was a decrease in stress levels and sleep disorders. Personal support also had a statistically significant negative correlation with stress (*τ*_b_ = −0.180, *p* = 0.003), sleep disorders (*τ*_b_ = −0.195, *p* = 0.003), and anxiety symptoms (*τ*_b_ = −0.147, *p* = 0.015). Thus, as personal support increased, there was a decrease in stress levels, anxiety symptoms, and sleep disorders. However, there was a positive correlation between risk support and stress (*τ*_b_ = 0.138, *p* = 0.020) and sleep disorders (*τ*_b_ = 0.142, *p* = 0.029), and anxiety symptoms (*τ*_b_ = 0.118, *p* = 0.047), all of which were statistically significant. Consequently, as risk-support scores increased, stress levels, sleep disorders, and anxiety symptoms also increased (Table 8).

**Table 8:** Kendall’s Tau-b correlation for dependent variables.

	PSS	PSQI	Work support	Personal support	Risk support	GAD-7
PSS	–					
PSQI (N^1^)	0.435^**^ 122	–				
Work support (N^1^)	−0.201^**^ 141	−0.168^*^ 123	–			
Personal support (N^1^)	−0.180^**^ 143	−0.195^**^ 123	0.400^**^ 143	–		
Risk support (N^1^)	0.138^*^ 143	0.142^*^ 123	−0.179^**^ 143	−0.075 145	–	

GAD-7 (N^1^)	0.517^**^ 143	0.444^**^ 123	−0.084 143	−0.147^*^ 145	0.118^*^ 145	–

GAD-7, Generalized Anxiety Disorder 7; PSQI, Pittsburgh Sleep Quality Index; PSS, Perceived Stress Scale. **Correlation is significant at the 0.01 level (2-tailed). *Correlation is significant at the 0.05 level (2-tailed). N^1^ varies because of missing data.

## Discussion

It is well known that physicians are exposed to a myriad of stressful situations on a daily basis because of the nature of their jobs. A pandemic such as COVID-19 can heighten the levels of stress to beyond what physicians are used to, putting those providers at a higher risk of developing psychological sequelae. The psychological effect of a pandemic on a population, as described in prior studies, can include the development of not only anxiety symptoms, stress, and depression but also physical manifestations, such as sleep disturbances (7, 26).

We were interested in evaluating the population of Puerto Rico, taking into consideration the psychological effect of a significant stressor. Prior to the pandemic, the residents of Puerto Rico had recently gone through several catastrophic natural disasters, including 2 major hurricanes and a series of earthquakes. Because of this recurrent exposure to a significant number of stressors in Puerto Rico over a short period of time, we aimed to evaluate the psychological impact of the pandemic on physicians working on the island and their perceptions of received support.

The prevalence obtained for anxiety symptoms was very similar to that of the reported data. Zhang et al., noted in their study in Iran, that there were high levels of anxiety in approximately 28% of the health care staff (9). Similarly, in a national USA survey performed during the COVID-19 pandemic, Young et al. reported that approximately 33% of the participating HCPs had shown significant symptoms of anxiety (9). Nevertheless, because of the relatively recent natural disasters that had assailed the island, we expected to find higher levels of moderate to severe anxiety symptoms in our population. After Hurricane Maria, a high psychological impact was seen in the Puerto Rican population, including those who later migrated to the mainland. Clinically significant symptoms were reported by Scaramutti et al. (2 years after the hurricane’s impact) and included anxiety (27%), posttraumatic stress disorder (44%), and depressive symptoms (33%) (15). When we compared our results, then, approximately 33.8% of our participants reported symptoms of anxiety that ranged from moderate to severe; there were no significant differences in the prevalence of our population compared with those of other studies (15). But if we compare our findings with Zhang et al.’s, a slight increase in the prevalence of anxiety symptoms is noted. Furthermore, when perceived stress was assessed, it was both significant and worrisome to find that around 69.9% of the participants had moderate to severe perceived stress. This contrasts with the findings of a 2020 study from Das et al., which revealed that 37.4 and 7.6% of their participants had moderate or severe stress, respectively (27). Similarly, a study from Almalki et al. a year later into the pandemic reported an estimated prevalence rate of stress among health care workers of 41.92%, almost during the same period of time of our study (28). That the stress levels of our physicians are so high, relatively speaking, is a cause for concern. It would be interesting to measure resilience in this group (comparing it with that of other populations), to determine whether it acts as a confounder and prevents the worsening of anxiety symptoms, despite the presence of higher levels of stress. Bozdağ and Ergün reported increases in psychological resilience levels in HCPs, and emphasized that increased life satisfaction, positive attitudes, and improved sleep quality were necessary if resiliency was to be augmented (29). In a qualitative study by Asayesh et al. evaluating the psychological experiences of physicians with COVID-19, interviewed participants referred their efforts to strengthen their hope, empathy and resilience to continue both their professional and personal life as adaptive emotional reactions (30). That being the case, we might assume that even if our physicians report high levels of stress, they may have coping strategies that promote the development of psychological resiliency.

Stress and anxiety have been strongly associated with sleep disorders, usually co-existing together. Insomnia is the most common type of sleep disorder reported in the population. In the majority of cases, it is not isolated but is associated with another medical or mental disorder (and is then classified as secondary insomnia) (13). High rates of sleep disorders during the pandemic were reported in physicians, as was described by Wang et al. in their study. They reported that 61.1% of the health care workers from the Hubei province in China had sleep disturbances when assessed with the PSQI scale. We found that physicians working in Puerto Rico during the pandemic had PSQI scores associated with poor sleep (a frequency of 67.9%), showing a high prevalence in this group of professionals. Analyzing the 7 components individually, the high prevalence of sleep disturbances and the reduced hours of sleep daily were noticeable. As mentioned previously, psychological disorders and insomnia are associated with impairment, disability, and alcohol and drug abuse, among other issues (31). This association is a major concern in the case of physicians who are managing life-threatening situations in their profession. Therefore, it is essential to develop strategies to provide support and identify professionals at risk if the development of disturbances that can affect patient care is to be minimized.

Furthermore, it is crucial that these individuals have strong support from their organizations. Physicians apart from concerns about long shifts or increased burden of work, were also constantly worried both that they and/or their families might become infected and that PPE might not be available (12). If they felt the tools or resources were not available through their leaders or organizations, they likely found themselves being exposed to another source of stress. Zhang et al. developed an instrument to measure perceived support in health care professionals working during the COVID-19 pandemic. In their study, adequate personal support predicted a decreased possibility of developing mild anxiety, and work support predicted a lower probability of moderate anxiety. Interestingly, our physicians in Puerto Rico reported a high perception of work support (65.7%) but a low perception of personal support (43.4%). If we were to analyze our results with Zhang’s interpretation of the scale, we would see that physicians in Puerto Rico are at a high risk of developing mild anxiety symptoms, which symptoms correlate with scores obtained for mild anxiety (26.9%). Zhang et al. did not find statistical significance between risk support and anxiety, but when we analyzed the Kendall’s Tau-b correlation, we found positive correlations between risk support and anxiety, sleep disorders, and stress that were statistically significant. Our sample reported low levels of risk support (37.2%), which is relevant, placing our physicians at a higher risk of anxiety, stress, and sleep disorders. Effective support from organizations is important if physicians are to be able to decrease the fear and uncertainty generated by a pandemic. Such support would have a beneficial effect on the well-being of the physicians and on the care they provide to their patients.

Our study had several limitations. The population surveyed was a small percentage of the total number of physicians in Puerto Rico; thus, our results cannot be generalized to the entire population of physicians on the island due to the small sample size. It is possible that some physicians never received the survey or had problems with their internet connection (necessary for the completion of the survey). During the last years, the increasing migration of physicians to the mainland due to natural disasters, the economic situation, and limited availability of programs for specialties and subspecialties on the island had a major impact in the quantity and the mean age of physicians in Puerto Rico. In a study reported in 2018, the median age of primary care physicians in Puerto Rico was 60 years, compared with 53 years nationally (32). Therefore, during the pandemic the majority of our physicians actively working were not non-young physicians when compared to other countries. The scarcity of physicians during that period is another limitation, including being non young physicians and the possible lack of expertise or confidence using electronic devices to complete the survey. Also, considering that physicians might have been working longer shifts because of the pandemic, the length of the survey may have been a limiting factor to its completion. Our study was conducted when the second wave of the pandemic was easing, and a large number of physicians may have been vaccinated. This could have affected the psychological status of the participants, who may have been more prepared for managing COVID-19 and were probably less frightened because they felt protected by the vaccine.

The COVID-19 pandemic left an impact on the entire population, including physicians. Puerto Rico entered the pandemic while still recovering from several natural disasters that had struck over the course of the previous several years. Given the nature of their jobs, physicians are at high risk of psychological disorders that may be potentiated by other stressors, such those provoked by a pandemic.

## Conclusion

Despite the study’s limitations, our findings present an overview of the prevalence of psychological disorders in physicians—which disorders are related to the impact of the COVID-19 pandemic—and will serve to increase our knowledge and awareness of this problem. Organizational support (as perceived by physicians during difficult times) may also have an impact on the development of anxiety disorders and psychological disturbances such as sleep problems and stress. Our data support the importance of organizational support and its correlation with the development of anxiety. Promoting the development of strategies to support physicians is vital to the identification of individuals at risk of experiencing psychological disturbances and the prevention of the same via the provision of timely interventions. It would be interesting to study resilience in this group of physicians to assess its relationship to the development of psychological disorders that are linked to the pandemic.

## Data availability statement

The original contributions presented in the study are included in the article/supplementary material, further inquiries can be directed to the corresponding author.

## Ethics statement

The study was reviewed and approved by the Institutional Review Board of the University of Puerto Rico, Medical Science Campus (protocol B2190620). The studies were conducted in accordance with the local legislation and institutional requirements. The participants provided their written informed consent to participate in this study.

## Author contributions

LS-P: Writing – original draft, Writing – review & editing. RG-DJ: Writing – review & editing. KM-G: Writing – review & editing. CA-A: Formal analysis, Methodology, Writing – review & editing. IA-R: Formal analysis, Methodology, Writing – review & editing.
